# Scan Quality and Entrance Skin Dose in Thoracic CT: A Comparison between Bismuth Breast Shield and Posteriorly Centered Partial CT Scans

**DOI:** 10.5402/2013/457396

**Published:** 2012-11-26

**Authors:** Rafel Tappouni, Bradley Mathers

**Affiliations:** ^1^Radiology Department, Penn State Hershey Medical Center, 500 University Drive, Hershey, PA 17033, USA; ^2^Internal Medicine Department, Penn State Hershey Medical Center, 500 University Drive, Hershey, PA 17033, USA

## Abstract

*Objectives*. To compare the effectiveness of the bismuth breast shield and partial CT scan in reducing entrance skin dose and to evaluate the effect of the breast shield on image quality (IQ). *Methods*. Nanodots were placed on an adult anthropomorphic phantom. Standard chest CT, CT with shield, and partial CT were performed. Nanodot readings and effective doses were recorded. 50 patients with chest CTs obtained both with and without breast shields were reviewed. IQ was evaluated by two radiologists and by measuring Hounsfield units (HUs) and standard deviation (SD) of HU in anterior subcutaneous region. *Results*. Breast shield and the partial CT scans reduced radiation to the anterior chest by 38% and 16%, respectively. Partial CT increased dose to the posterior chest by 37% and effective dose by 8%. Change in IQ in shield CT was observed in the anterior chest wall. Significant change in IQ was observed in 5/50 cases. The shield caused an increase of 20 HU (*P* = 0.021) and a 1.86 reduction in SD of HU (*P* = 0.027) in the anterior compared to posterior subcutaneous regions. *Summary*. Bismuth breast shield is more effective than the partial CT in reducing entrance skin dose while maintaining image quality.

## 1. Introduction

Computed tomography (CT) has emerged as an important diagnostic tool in clinical medicine. Its use has grown exponentially over the years, rising from 3 million in 1980 to 67 million in 2006, an equivalent of a 600% increase from 1980 to 2006 [1]. This significant rise in frequency also brings into question the level of radiation exposure to patients. 

The potential carcinogenic effect of this increased radiation dose on radiosensitive tissues has fostered much concern recently [2] and breast tissue exposure from chest CT is an area of particular concern in females. In 2008, the International Commission of Radiation Protection (ICRP) increased the tissue weighting factor for the breast from 0.05 to 0.12 [3]. The radiation exposure to the breast from a chest CT is estimated to be 2.0–3.5 rad, which is equivalent to 10 mammograms or 100 chest radiographs [4]. These doses far exceed the American College of Radiology recommendation of 0.3 rad or less for a standard 2-view mammography. The delivery of 1 rad to a woman younger than age 35 years old is estimated to increase her lifetime risk of breast cancer by 13.6% [4–7]. Due to this risk, several techniques have been developed to reduce the radiation exposure to the breast during a chest CT. 

The in-plane bismuth breast shield has been shown to be effective at reducing radiation dose to the breast. An early study showed a 57% reduction in radiation dose to the breast [4]. Subsequent studies have confirmed these findings, with radiation dose reduction ranging from 26% to 41% [8–13]. Phantom studies have found that the breast shield does not cause a significant change in lung image quality, although it may impair image quality in the breast tissue in phantom studies [8, 9, 11, 13]. The superficial structures of the breast tissue, however, are rarely of diagnostic concern in a CT examination. To date, no adult patient study or qualitative assessment of the entire chest with the breast shield has been conducted. 

**Figure 1:**
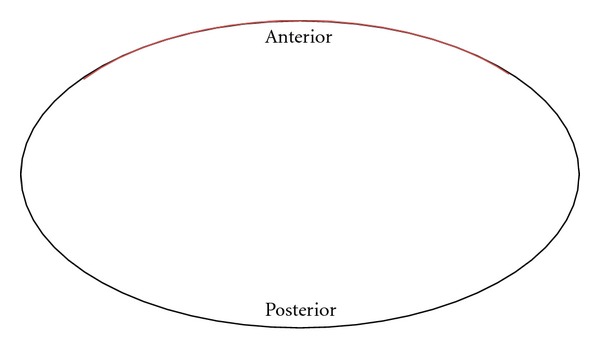
Illustration of technique utilized in posteriorly centered partial CT scan. The tubes are switched on during 323° out of 360° per rotation. The radiation intensity is then reduced along the remaining 37° (red line) corresponding to the breast region.

Posteriorly centered partial CT scanning is a newer technique in which the radiation intensity is reduced in the anterior chest region. This technique avoids exposing the breast directly as the tube output is reduced along the anterior 37° corresponding to the breast region and then switch on during posterolateral 323° out of 360° per rotation and is then (Figure 1). Only one study has previously examined the effectiveness of the partial CT scan and found that the partial CT scan reduced radiation dose to the breast by 50%, similar to that of the bismuth breast shield [14]. However, the partial CT scan did not cause an increase in noise or impairment of image quality as seen by the breast shield in this phantom study.

The first goal of this study is to compare the effectiveness of the bismuth breast shield and partial CT scan in reducing entrance skin dose in anthromorphic phantom subjects. Assuming that very little attenuation occurs in the breast tissue, the entrance skin dose is an accurate measurement of the radiation dose to the breast. The second goal of this study is to examine the qualitative and quantitative effects of the breast shield on CT image quality in adult human subjects.

## 2. Methods and Materials

### 2.1. Anthropomorphic Phantom Study Comparing Entrance Skin Dose Posteriorly Centered Partial CT with Breast Shield

**Figure 2:**
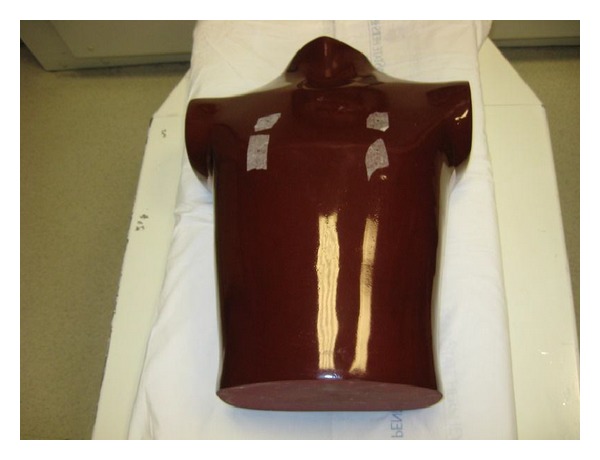
Image of adult anthropomorphic torso phantom (RS 310 lung/chest phantom, Fluke Biomedical) used.

**Figure 3:**
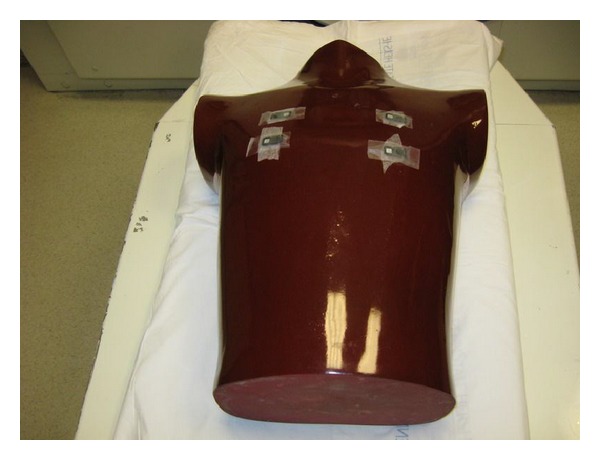
Image of adult phantom with four nanodots (Landauer, Inc., Glenwood, IL) placed on the anterior surface corresponding to the breast region.

**Figure 4:**
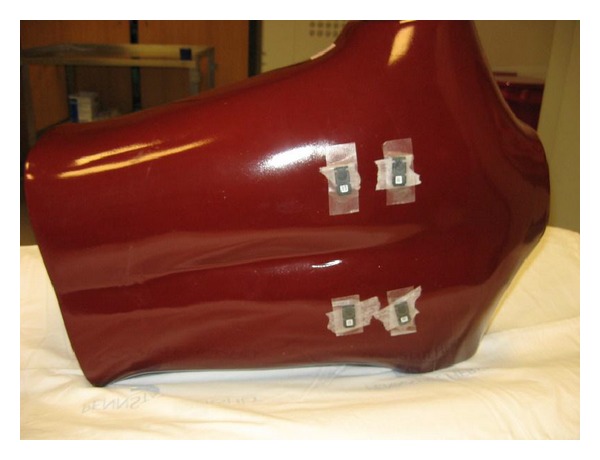
Image of adult phantom with four nanodots (Landauer, Inc., Glenwood, IL) placed on the posterior surface.

An adult anthropomorphic torso phantom (RS 310 lung/chest phantom, Fluke Biomedical) was used and four areas were marked with tape on anterior and posterior surfaces (Figure 2). A topogram was obtained once to plan scanning area to include only the phantom. Four nanoDots (Landauer, Inc., Glenwood, IL), which use optically stimulated luminescence technology, were placed on the anterior chest surface (Figure 3) and another four were placed on the posterior surface (Figure 4). 

The phantom was scanned using the parameter clinically used for adult chest CT protocol (256-MDCT, Siemens Medical solutions, Malvern, PA) consisting of 120 kVp, 0.5-second rotation time, 1.2 pitch, 128 × 0.6 mm effective collimation. Tube current modulation was used with reference mA of 110.

**Figure 5:**
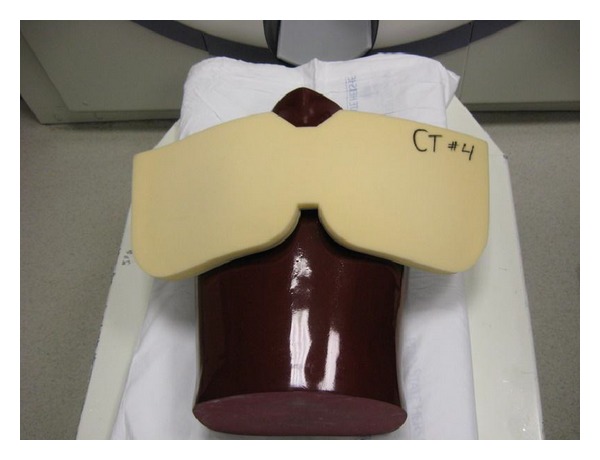
Image of anthropomorphic phantom with bismuth breast shield (AttenuRad Radiation Protection; F & L Medical Co., Vandergrift, PA; 0.060 mm Pb equivalent) placed on anterior surface on top of padding.

After substituting the nanoDots with a new set a second scan was obtained using same parameters after placing a bismuth breast shield(AttenuRad Radiation Protection; F & L Medical Co., Vandergrift, PA; 0.060 mm Pb equivalent) on anterior surface covering the four anterior TLDs (Figure 5). Care was taken during replacement of nanoDots not to change the overall position of the phantom.

A third scan was obtained after placing a new nanoDot set using posteriorly-centered partial CT protocol, which avoids exposing the breast directly as the tube switches on during 323° out of 360° per rotation. The scan parameters consisted of 120 kVp, 0.28-second rotation time, 0.6 pitch, 128 × 0.6 mm effective collimation using tube current modulation with reference mA of 110. The readings from the three sets of 8 nanoDots were recorded inaddition to effective dose, CT dose index (CTDI), and dose length product (DLP) for each scan.

### 2.2. Patient Study Assessing CT Image Quality and Noise with Breast Shield in Place

 This retrospective study was approved by the institutional review board and was in compliance with HIPAA regulations and informed consent was waived. Between December 2008 and February 2009, 50 female patients with chest CTs performed for clinical indications obtained both with and without breast shields within a 12-month period were selected. The shield used was a commercially available AttenuRad CT Breast Shield System (F & L medical products, Vandergrift, PA). The breast shield was applied after topogram using 16-, 40-, 64-slice Siemens multidetector scanners protocol (256-MDCT, Siemens Medical solutions, Malvern, PA). The scan protocol was 120 kVp, 0.75 mm collimation, 3-mm slice thickness, 3-mm reconstruction interval, with images viewed in mediastinum and lung windows.

### 2.3. Qualitative Assessment

**Figure 6:**
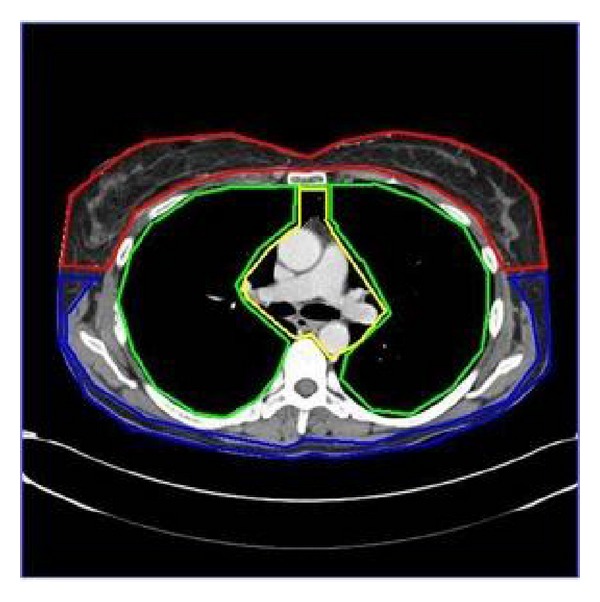
Axial contrast CT image of chest. Image delineates the six regions used in the qualitative comparison of the scans with and without the breast shield in patients. The six regions are anterior thoracic wall (red), lateral and posterior wall (blue), lung parenchyma [10], mediastinum, heart, and aorta and great vessels (all three within yellow).

 The chest was first divided into six areas: anterior thoracic wall, lateral and posterior wall, aorta and great vessels, mediastinum, heart, and lung parenchyma (Figure 6). Two chest radiologists, each with 10 years experience, then compared the image quality of scans with and without the breast shield in regards to these six areas. Each area was evaluated with the following score criteria:

 Score 1 for no difference in scan quality;
 Score 2 for minor change in image quality of no clinical significance;
 Score 3 for a significant change in image quality.

### 2.4. Quantitative Assessment

Image quality and noise in scans with and without the breast shield was also performed. Image quality was evaluated in regards to the Hounsfield units (HU) in the anterior and posterior subcutaneous fat. Noise was measured as the standard deviation of HU in the region of interest in the anterior and posterior subcutaneous fat.

### 2.5. Statistical Analysis

 A paired *t*-test (parametric) was performed comparing means between dependent groups (anterior versus posterior) in the quantitative assessment of the patient study. The outcome variables being compared between anterior and posterior groups were HU and standard deviation. For all tests, *P* values of less than 0.05 were considered to indicate a significant difference. Interobserver variability regarding the qualitative assessment of the CT image in the patient study was assessed with *κ* statistics.

## 3. Results

### 3.1. Anthropomorphic Phantom Study Comparing Posteriorly Centered Partial CT with Breast Shield

 The results of the phantom study are shown below in Tables 1 and 2.  Table 1 contains the readings from the eight individual nanoDots for the three runs comparing standard CT, standard CT with breast shield, and partial CT.  Table 2 summarizes the findings of the study. The lowest average radiation dose to the anterior nanoDots was seen with the CT with the breast shield (426 mrad), as compared to CT without the breast shield (686 mrad) and partial CT (590 mrad). When compared with the standard CT alone, the breast shield reduced radiation dose to the anterior nanoDots by 38% ((686−426)/686) and the partial CT scan reduced radiation dose to the anterior nanoDots by 16% ((686−590)/686). The highest DLP, CTDI, average effective dose, and average radiation dose to the posterior nanoDots were observed in the partial CT scan (331 mGy-cm, 6.47 mGy, 95 mAs, 826 mrads, resp.). When compared to the standard CT alone, the partial CT scan increased the average dose to the posterior nanoDots by 37% ((826−602)/602), the average effective dose by 8% ((95−88)/88), the CTDI by 8% ((6.47−5.99)/5.99), and the DLP by 4% ((331−318)/318). When compared to the standard CT alone, no significant difference was observed in standard CT with the breast shield compared to standard CT in respect to average radiation dose to the posterior nanoDots (0.7% increase (606−602)/602)), average effective dose (1.1% decrease (88−87)/88)), CTDI (1.0% decrease (5.99−5.93)/5.99)), and DLP (0.9% decrease (318−315)/318). 

### 3.2. Patient Study Assessing CT Image Quality and Noise with Breast Shield

#### 3.2.1. A Qualitative Assessment

 In all 50 cases, no difference (score 1) in scan quality was seen in the lateral and posterior chest wall, aorta and great vessels, mediastinum, heart, and lung parenchyma. There was no discordance between the two readers (*κ* = 1.00, near perfect interobserver agreement) for those five areas. In 27 of 50 patients, both readers observed no difference (score 1) in scan quality in the anterior chest wall, which leaves 23 patients. In 9 of the 23, both readers observed a minor change of no clinical significance (score 2) in scan quality in the anterior chest wall. 

**Figure 7:**
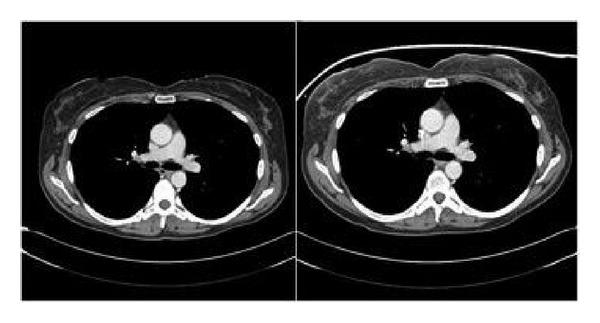
Axial contrast CT scans of the chest. This is an example of a CT scan comparison in which discordance was present between the two readers. The image on the left is a CT with breast shield, while the image on the right is without breast shield. In this case, one reader gave a score 1 while the other gave score 3.

 Some discordance between the two readers was observed in the remaining 14 patients related to the anterior chest wall, with a *κ* value of 0.36 (fair interobserver agreement, *P* < 0.002). The five patients given score 3 were discordant between the two readers. There were nine patients with scores of 2 and 1, three patients with scores of 3 and 2, and two patients with scores of 3 and 1. An example of this discordance is shown in Figure 7. There were no cases in which both readers observed a significant change in image quality (score 3). 

#### 3.2.2. Quantitative Assessment

 The results of the quantitative assessment of scan quality with the breast shield are summarized in Table 3. The mean HU in the posterior subcutaneous fat in patient CT scans using breast shield was −81.91 HU ± 55.90 (standard deviation) while the mean HU in the anterior subcutaneous fat was −62.34 HU ± 23.00. Thus, the breast shield caused a significant increase of approximately 20 HU in the anterior subcutaneous fat as compared to the posterior (*P* = 0.021). It is important to note that HU in the posterior subcutaneous fat is 0–10 HU lower than anterior subcutaneous fat due to CT table attenuation. The shield caused a 1.86 reduction in SD of the HU in the anterior compared to posterior subcutaneous regions (*P* = 0.027). The mean SD of HU was 12.98 ± 11.64 in the anterior and 14.84 ± 14.58 in the posterior subcutaneous fat regions.

## 4. Discussion

 A recent major goal has been to reduce the amount of radiation to the anterior chest during a CT scan without compromising image quality of deeper structures. Many of the factors that determine radiation dose are under the control of the radiologist, such as number of scans, tube current, scanning time, axial scan range, scan pitch, and tube voltage [15]. The placement of external barriers, such as the bismuth breast shield, has been in use for many years and several studies have confirmed its effectiveness in reducing breast radiation dose. However, no patient study has been performed demonstrating the effect of breast shield on image quality. More recently, a posteriorly-centered partial CT scan has become commercially available. Its effectiveness at breast radiation reduction is not well established, and thus further studies are needed.

 The first aspect of our study compared the breast shield and the partial CT in reducing entrance skin dose in an anthromorphic phantom. We found that while both techniques reduced dose compared to the standard CT with current modulations, the breast shield had a significantly greater reduction in entrance dose (38%) than the partial CT scan (16%). Furthermore, the partial CT scan caused a significant increase in the average dose to the posterior nanoDots (37%), average total effective dose (8%), CTDI (8%), and DLP (4%). Thus, our study indicates that the breast shield is superior to the partial CT scan in reducing entrance skin dose during a CT examination. The breast shield causes a greater decrease in radiation than the partial CT scan and does not increase radiation to the other tissues within the thorax. 

 The increased radiation observed with the partial CT scan is due to the compensatory increase of the mA and therefore dose along the posterolateral 323° to compensate for the reduced dose along the anterior 37°. This compensation occurred in order to maintain image quality; as a result higher mA and radiation are produced to the lateral and posterior chest. While the other thoracic tissues may not be as radiosensitive as the breast, a long-term increase in radiation to these tissues should be avoided, as negative consequences would be more likely.

 Of note is the difference in dose between the superior and inferior nanoDots in all three scans. The superior readings were consistently higher than the inferior readings. This is due to the spiraling effect of the CT beam.

 After determining that the breast shield was more effective than the partial CT in reducing entrance skin dose, the assessment of the clinical cohort was undertaken to further assess the breast shield's effect on image quality. In the qualitative assessment, no difference in image quality was observed in the lateral and posterior chest wall, aorta and great vessels, mediastinum, heart, and lung parenchyma. Although discordance between the two readers was present in regards to the anterior chest wall, in a majority of cases no significant change in image quality was observed. 

 In the quantitative assessment, the breast shield resulted in a significant increase of 20 HU and a minor reduction in SD of HU as a measure of noise in the anterior subcutaneous fat as compared to the posterior subcutaneous fat. Although a control was not present in this study, a difference of 20 HU between the anterior and posterior fat is more than is typically seen in a standard chest CT, which normally ranges between 0–10 HU. The reduction in the SD is likely attributed to beam hardening from the breast shield, which leads to a more uniform beam. 

In both quantitative and qualitative assessment, no change in image quality was noted in any areas except the anterior chest wall. Although some differences in image quality were noted in the anterior chest wall, this is of minimal significance considering that the area is rarely of diagnostic concern in a chest CT. Therefore, the breast shield is able to reduce entrance skin dose without compromising image quality of structures of diagnostic importance.

 The results from our study compare favorably with those of previous studies. The 38% reduction in entrance skin dose observed with the breast shield falls within the 26% to 57% range seen in other studies [4, 8–13]. Our study confirmed that the breast shield does not compromise image quality of the lungs, as reported in previous phantom studies [8, 9, 11, 13]. An early study noted an increase in artifact in the area directly under the breast shield when placed directly on the skin [4]. This was prevented in our study by the foam, which is part of the shield, between the skin and the bismuth, reducing the amount of scatter radiation entering the patient.

 Both agreement and discordance were present between our study and the study performed by Vollmar and Kalender [14]. Similar to our study, their study found a 50% dose reduction while using the breast shield. However, they reported a 40% increase in noise and impaired image quality of the lungs and heart due to artifacts with the breast shield. It is important to note that no padding was placed between the skin and the shield in their study, which may have caused increased scatter radiation and thus impaired image quality and increased noise. In addition, their study measured image quality with phantom scans while we used patient scans. The difference in subjects may have contributed to the noted discordance, and our patient study is more clinically applicable.

In regards to the partial CT, they observed a 50% dose reduction, as compared to the 16% reduction seen in our study. This difference is likely due to the use of a simulated partial CT in their study compared to a commercially available partial CT used in our study. The authors tried to simulate the partial CT by using only 323° of the acquired raw data from a standard CT. Therefore, there was zero direct anterior radiation in the chest. The partial CT scan used in our study reduces its dose to a minimum during the anterior 37° but does not turn off. This additional radiation exposure present in our study may have decreased the level of reduction observed with the partial CT in our study as compared to their study. Their study also observed a 15–20% increase in radiation dose to the spine with the partial CT. To achieve the same level of exposure as the standard CT, they increased the intensity of the tube current. Thus, both studies observed an increase in radiation dose to the spine.

Several limitations exist in our study. First, we did not measure the radiation dose within the breast tissue itself, but rather chose to measure the skin entrance dose; however, we believe this is the most critical aspect of radiation exposure to the breast. Second, we did not investigate the image quality in partial CT scanning. However, this technique is the manufacturer recommended default setting for all adult chest CTs and the quality of scanning is identical to standard chest CT. Third, there was no control in the patient study for the quantitative assessment of CT image quality with the breast shield. The HU and standard deviation of HU was not recorded in the posterior subcutaneous fat and therefore no control was available. 

In conclusion, bismuth breast shield is more effective than the partial CT in reducing entrance skin dose without increasing the dose to other areas within the thorax. Furthermore, the breast shield maintained the overall CT image quality. Although the partial CT reduced entrance skin dose, it increased the average effective dose to the remaining thoracic tissues, particularly the posterior chest wall. With the increasing use of CT examinations and a goal to reduce radiation to the breast, radiologists need to take charge of radiation dose. Radiologists should use the breast shield in every female chest CT. 

## Figures and Tables

**Table 1:** Results from individual nanodots in the anthromorphic phantom study comparing partial CT with breast shield.

Position on phantom	Right anterior	Left anterior	Right posterior	Left posterior
Nanodot Doses (mrad) with Standard CT alone				

Superior	842	804	614	682
Inferior	517	582	546	566

Nanodot Doses (mrad) with Standard CT + Breast Shield				

Superior	476	518	624	675
Inferior	353	358	573	553

Nanodot Doses (mrad) with Partial CT				

Superior	738	663	914	917
Inferior	514	447	721	751

**Table 2:** Summary of findings from anthromorphic phantom study comparing partial CT with breast shield.

Position	NanoDot Avg Anterior (mrad)	NanoDot Avg Posterior (mrad)	Avg Effective Dose (mAs)	CTDI (mGy)	DLP (mGy-cm)
Standard CT alone	686	602	88	5.99	318
Standard CT + breast shield	426	606	87	5.93	315
Partial CT	590	826	95	6.47	331

CTDI: CT dose index. DLP: Dose length product.

**Table 3:** Summary of quantitative assessment of CT image quality with breast shield. Hounsfield units and standard deviation are compared in the anterior and posterior subcutaneous fat. Values are in Hounsfield units.

Location	Mean	Standard deviation	95% Confidence Interval for Mean	Paired *t*-Test *P* value
Anterior HU	−62.34	23.00	(−68.81, −55.87)	0.021
Posterior HU	−81.91	55.90	(−97.64, −66.19)	

Anterior SD	12.98	11.64	(9.71,16.26)	0.027
Posterior SD	14.84	14.58	(10.74,18.94)	
